# Mitochondrial C1-FDX Depletion Perturbs ROS Homeostasis and Iron Metabolism in Arabidopsis

**DOI:** 10.3390/ijms27209349

**Published:** 2026-10-21

**Authors:** Manna Huang, Kexin Zhang, Tingting Wang, Peiyi Li, Yue Xin, Baoyin Chen

**Affiliations:** National Key Laboratory of Wheat Improvement, College of Agronomy, Shandong Agricultural University, Tai’an 271018, China

**Keywords:** mitochondria, ROS, NADH/NAD^+^ homeostasis, iron homeostasis, ferredoxin, Arabidopsis

## Abstract

Mitochondrial complex I (CI) is a major source of reactive oxygen species (ROS) in plant cells, and iron–sulfur (Fe-S) cluster proteins are critical for maintaining CI structure and electron transport. C1-FDX, a mitochondrion-localized ferredoxin containing a [2Fe-2S] cluster, acts as a structural component and assembly factor of mitochondrial CI and complex V (CV) in Arabidopsis. In this study, we further characterized the physiological and molecular functions of C1-FDX in regulating ROS production, NADH/NAD^+^ homeostasis, and iron metabolism. Histochemical staining demonstrated that loss of function of C1-FDX in Arabidopsis *c1-fdx* mutants accumulated excessive superoxide anions (O_2_^•−^) but exhibited reduced hydrogen peroxide (H_2_O_2_) relative to the wild type (WT). Biochemical assays further confirmed that there was an elevated NADH/NAD^+^ ratio in mutant seedlings, which facilitated O_2_^•−^ overproduction at mitochondrial CI. Loss of C1-FDX also led to Fe^3+^ accumulation in mutant tissues. Immunoblot and qRT-PCR results showed that the abundance of MnSOD markedly increased, while other antioxidant proteins, including FeSOD, CAT1, and GR, were downregulated in *c1-fdx* mutants of Arabidopsis. The enhanced MnSOD activity accelerated the conversion of O_2_^•−^ into H_2_O_2_, and the subsequently accumulated Fe^3+^ may be derived from the Fenton reaction, which consumes H_2_O_2_, ultimately resulting in decreased H_2_O_2_ levels. Additionally, deficiency of C1-FDX disturbed the abundance of mitochondrial chaperones, proteases, and alternative respiratory pathway proteins. Collectively, our findings demonstrate that C1-FDX participates in the regulation of mitochondrial ROS balance and iron homeostasis and suggest there is a novel regulatory link between ferredoxin, the electron transport chain, and the Fenton reaction in plant mitochondria.

## 1. Introduction

Mitochondria are semi-autonomous organelles universally existing in eukaryotic cells. They undertake the core task of energy metabolism and participate in numerous vital biological processes, including cell signal transduction, programmed cell death, ion homeostasis, and metabolite biosynthesis [1,2]. The majority of cellular ATP is synthesized by the OXPHOS system embedded in the inner mitochondrial membrane (IMM). The OXPHOS system consists of five large protein complexes, namely, NADH:ubiquinone oxidoreductase (CI), succinate dehydrogenase (Complex II, CII), cytochrome *c* reductase (Complex III, CIII), cytochrome *c* oxidase (Complex IV, CIV), and ATP synthase (CV) [3,4]. In the classic electron transport chain (ETC), electrons derived from the oxidation of NADH and FADH_2_ are sequentially transferred through CI, CIII, and CIV [5]. During electron translocation, CI, CIII, and CIV pump protons from the mitochondrial matrix across the inner mitochondrial membrane into the intermembrane space, forming a transmembrane proton electrochemical gradient [5,6,7,8,9]. The potential energy stored in this gradient is subsequently utilized by CV to catalyze ADP phosphorylation and generate ATP [10].

During electron transfer along the respiratory chain, partial electrons leak and react with molecular oxygen to produce ROS, including O_2_^•−^, H_2_O_2_, and the hydroxyl radical [2]. Under physiological conditions, low concentrations of ROS act as important signaling molecules to regulate plant growth, development, and stress responses [7,11,12]. However, excessive ROS accumulation will cause oxidative damage to intracellular biomolecules, including DNA, RNA, proteins, and membrane lipids, and even trigger cell death [2]. In plant and mammalian mitochondria, approximately 70% of total cellular ROS are produced by the ETC, and CI and CIII are the two dominant sites for O_2_^•−^ generation [1,13]. Accumulating evidence has proven that CI accounts for the largest proportion of O_2_^•−^ production among all respiratory complexes [14,15]. Two distinct O_2_^•−^ production sites have been identified within plant mitochondrial CI: the NAD pool site and the ubiquinone (Q) pool site [16]. The redox state of the NADH/NAD^+^ pool is a decisive factor regulating O_2_^•−^ production at CI. A decreased intracellular NAD^+^ level leads to an elevated NADH/NAD^+^ ratio, which greatly promotes electron leakage and O_2_^•−^ formation [16]. Mechanistically, only the nucleotide-free state of reduced flavin groups in CI can interact with O_2_ to generate O_2_^•−^, which explains the close correlation between NADH/NAD^+^ redox status and the ROS output of CI [9,17].

Once produced in the mitochondrial matrix, O_2_^•−^ is rapidly catalyzed by manganese superoxide dismutase (MnSOD), a matrix-localized antioxidant enzyme, to form H_2_O_2_ [18] (Figure S1). Unlike O_2_^•−^, H_2_O_2_ has a relatively longer half-life and stronger membrane permeability, and it can diffuse to multiple subcellular compartments to participate in signal regulation [11]. Nevertheless, H_2_O_2_ can further react with ferrous ions (Fe^2+^) via the classic Fenton reaction to generate hydroxyl radicals (·OH), the most reactive ROS in living organisms [19,20] (Figure S1). The diffusion distance of ·OH in the cellular micro-environment is only about 3 nm, which is equivalent to the average diameter of a single protein molecule. Therefore, ·OH can instantly oxidize and damage surrounding proteins, lipids, and nucleic acids, resulting in severe oxidative injury to mitochondria and even the whole cell [21]. To maintain intracellular redox homeostasis, plants have evolved a sophisticated ROS scavenging system composed of enzymatic and non-enzymatic antioxidants. The enzymatic components include SOD family proteins, catalase (CAT), glutathione reductase (GR), and peroxidases; the non-enzymatic antioxidants mainly include glutathione (GSH), ascorbic acid, and flavonoids [21]. Although the basic metabolic pathways of mitochondrial ROS have been clarified, the upstream regulatory factors that coordinate ROS production, scavenging, and the Fenton reaction in plant mitochondria remain poorly understood.

Fe-S clusters are ubiquitous prosthetic groups in organisms. They are widespread in redox enzymes, electron carriers, and metabolic enzymes. Ferredoxins (FDXs) constitute a typical class of low-molecular-weight Fe-S proteins that generally carry [2Fe-2S] clusters [22]. Owing to the reversible redox transition between the Fe^2+^ and Fe^3+^ states of iron atoms, FDXs possess strong electron transfer capacity and participate in a variety of intracellular redox reactions [23]. In addition to acting as electron shuttles, FDXs also serve as key assembly factors for the biogenesis of Fe-S-cluster-containing proteins [24]. Mitochondrial CI contains five core subunits equipped with Fe-S clusters, including NDUFV1, NDUFV2, NDUFS1, NDUFS7, and NDUFS8 [5]. These Fe-S subunits constitute the core active region for NADH oxidation and electron transfer regarding CI [5,25]. Numerous studies have focused on the structure and function of these Fe-S subunits, while the ferredoxin proteins that regulate the stability, assembly, and activity of CI Fe-S subunits have not been systematically studied.

C1-FDX (Complex I Ferredoxin) is a recently identified mitochondrion-targeted [2Fe-2S] ferredoxin in plants. Structural biology and biochemistry studies have revealed that C1-FDX has two essential functions in plant mitochondria. Firstly, C1-FDX serves as a core structural component of CI, forming a unique ferredoxin bridge domain that connects the ubiquinone reduction module (Q module) and γ-carbonic anhydrase domain (CA domain) of CI in *Arabidopsis thaliana*, green algae, and leguminous plants [4,26,27]. Secondly, C1-FDX participates in the assembly of monomeric CI, CV, and the CI+CIII_2_ supercomplex during the biogenesis of mitochondrial respiratory complexes [8,28]. Genetic evidence has shown that loss-of-function mutation of C1-FDX in Arabidopsis leads to retarded seed germination in the early growth stage and reduces the abundance and catalytic activity of CI and CV subcomplexes [8]. A recent assembly intermediate analysis further confirmed that C1-FDX is integrated into the 400 kDa membrane arm intermediate of CI at the early assembly stage [9]. Despite the progress noted above, the physiological roles of C1-FDX in regulating mitochondrial redox metabolism, iron homeostasis, and ROS-related reactions remain elusive.

In this study, we adopted two independent *c1-fdx* knockout mutant lines of Arabidopsis as research materials [8] and combined histochemical staining, biochemical quantification, immunoblotting, and qRT-PCR analysis to explore the function of C1-FDX in mitochondrial ROS metabolism, NADH/NAD^+^ balance, and iron ion accumulation. We found that *c1-fdx* mutants showed increased O_2_^•−^ levels, decreased H_2_O_2_ levels, elevated NADH/NAD^+^ ratios, and iron accumulation. Further molecular evidence proved that C1-FDX regulates the expression of mitochondrial antioxidant proteins and mediates the Fenton reaction to remodel ROS homeostasis. Our research clarifies the molecular mechanism of C1-FDX in linking ETC function, ROS metabolism, and iron homeostasis and provides a theoretical basis for further understanding the physiological functions of plant mitochondrial ferredoxins and respiratory complex I.

## 2. Results

### 2.1. Loss of C1-FDX Induces Opposite Changes in O_2_^•−^ and H_2_O_2_ Accumulation

**Figure 1:**
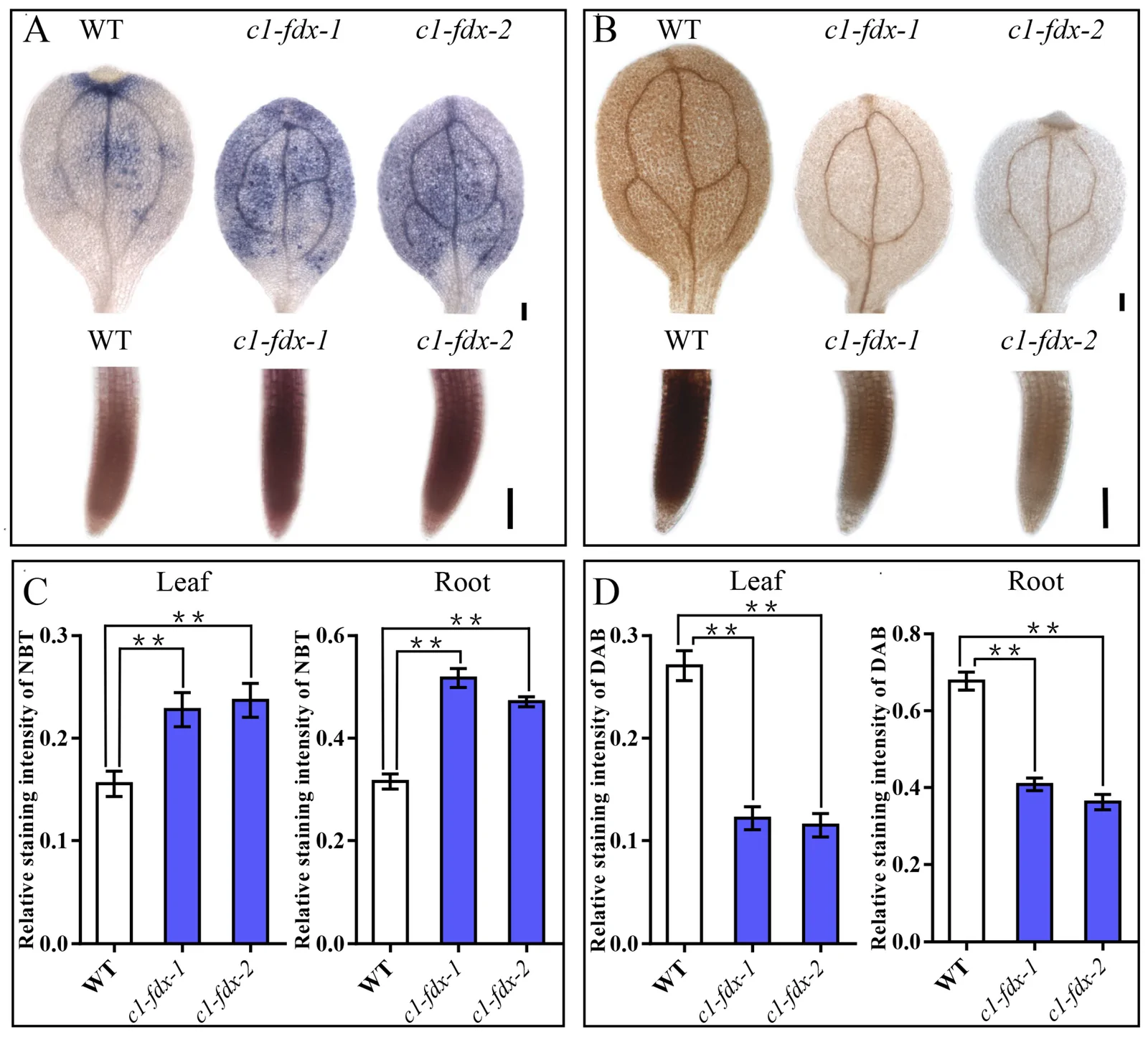
**ROS levels in Arabidopsis *c1-fdx* mutants.** Wild-type and *c1-fdx* mutant lines in Arabidopsis were grown under normal conditions for 3 days after stratification. The leaves and roots were stained for O_2_^•−^ (Nitroblue tetrazolium, NBT) (**A**) and H_2_O_2_ (3,3′-Diaminobenzidine, DAB) (**B**). Scale bars: 250 μm (leaves); 100 μm (roots). Relative staining intensities of NBT (**C**) and DAB (**D**), calculated from (**A**) and (**B**), respectively. Data are means ± SDs of three biological replicates. The wild type and each mutant were compared using one-way ANOVA followed by Dunnett’s test. Asterisks indicate significant differences relative to the WT plants. ** *p* < 0.01.

To explore the effect of C1-FDX deficiency on mitochondrial ROS metabolism, we performed NBT and DAB histochemical staining to detect the in situ levels of O_2_^•−^ and H_2_O_2_ in three-day-old WT, *c1-fdx-1*, and *c1-fdx-2* seedlings. NBT-staining results showed that the leaves and roots of the two *c1-fdx* mutant lines presented obvious deep-blue staining, while the WT seedlings only showed weak background staining (Figure 1A,C). This phenotype indicated that the O_2_^•−^ content had significantly increased in the *c1-fdx* mutants relative to the WT plants. On the contrary, DAB staining for H_2_O_2_ detection showed completely different results. The brown staining intensity in the leaves and roots of *c1-fdx* mutants was obviously lower than that for the WT, proving that H_2_O_2_ content markedly reduced after the loss of function of C1-FDX (Figure 1B,D).

### 2.2. Elevated Mitochondrial NADH/NAD^+^ Ratios Account for Excessive O_2_^•−^ Production in c1-fdx Mutants

**Figure 2:**
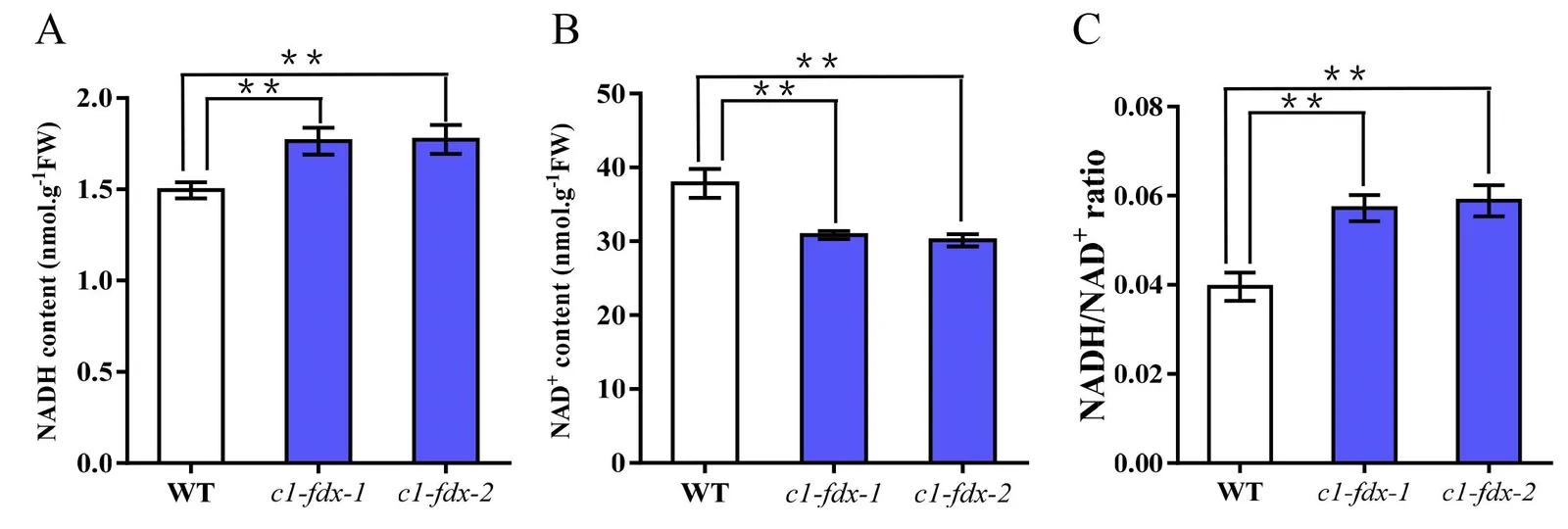
**Mitochondrial NADH and NAD^+^ levels in Arabidopsis *c1-fdx* mutants.** (**A**) NADH content, (**B**) NAD^+^ content, and (**C**) NADH/NAD^+^ ratios. Data are means ± SDs of three biological replicates. The wild type and each mutant were compared using one-way ANOVA followed by Dunnett’s test. Asterisks indicate significant differences relative to the WT plants. ** *p* < 0.01.

The redox state of the intracellular NAD pool is a key factor controlling O_2_^•−^ production at mitochondrial CI [16,17]. To clarify the molecular cause of O_2_^•−^ accumulation in *c1-fdx* mutants, we quantitatively detected the mitochondrial NADH and NAD^+^ content of three-day-old seedlings. Biochemical assay results showed that the NADH content in *c1-fdx-1* and *c1-fdx-2* was significantly higher than that in the WT (Figure 2). Correspondingly, the NAD^+^ content of the mutant lines decreased remarkably relative to the WT (Figure 2).

Further calculation of the NADH/NAD^+^ ratio showed that the ratio in *c1-fdx* mutants increased sharply, reaching an extremely significant difference level relative to the WT (Figure 2). A high NADH/NAD^+^ ratio can increase the probability of electron leakage from CI and promote the reduction of O_2_ to generate O_2_^•−^ [16,17]. Considering the ROS-staining results in tandem, we concluded that the C1-FDX dysfunction disrupted normal electron transport for CI, disturbed the circulation balance of NADH and NAD^+^, and finally led to the overproduction of O_2_^•−^ in mutant seedlings.

### 2.3. Iron Ions Accumulate in c1-fdx Mutant Seedlings

**Figure 3:**
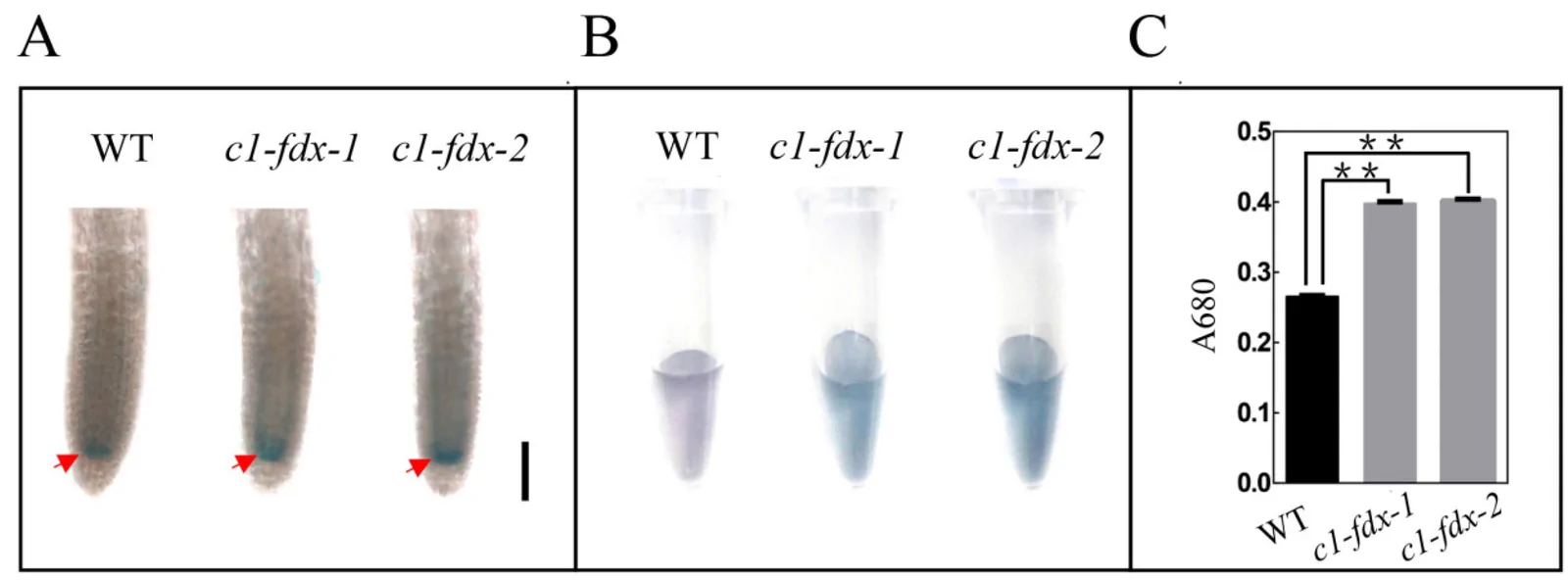
**Fe^3+^ levels in Arabidopsis *c1-fdx* mutants.** Wild-type and *c1-fdx* mutant lines in Arabidopsis were grown under normal conditions for 3 days after stratification. (**A**) The roots of the seedlings were treated with Prussian blue staining to assess the Fe^3+^ content. The leachate was photographed (**B**) and its absorbance (A680) was measured (**C**) following the Prussian blue staining process. Red arrows indicate the Fe^3+^ enriched region. Scale bars, 100 μm. Data are means ± SDs of three biological replicates. The wild type and each mutant were compared using one-way ANOVA followed by Dunnett’s test. Asterisks indicate significant differences relative to the WT plants. ** *p* < 0.01.

As a typical [2Fe-2S] ferredoxin, mammalian mitochondrial FDXs are closely associated with intracellular iron homeostasis and Fe-S cluster biogenesis [23,24,29]. To verify whether plant C1-FDX has similar functions, we used Prussian blue staining to detect Fe^3+^ distribution in seedling roots. The staining results showed that the Fe^3+^ staining intensity at the root tips of *c1-fdx* mutants was significantly higher than that of the WT (Figure 3). Meanwhile, the Fe^3+^ content in the leaching solution for the mutant tissues was also significantly higher (Figure 3). These results prove that loss of C1-FDX leads to obvious Fe^3+^ accumulation in Arabidopsis seedling tissues. The accumulated Fe^3+^ might be derived from the Fenton reaction in mutant cells (Figure S1). This might be one of the factors in the reduction of H_2_O_2_ in *c1-fdx* mutants.

### 2.4. C1-FDX Mutation Remodels the Expression of Mitochondrial Antioxidant Proteins

**Figure 4:**
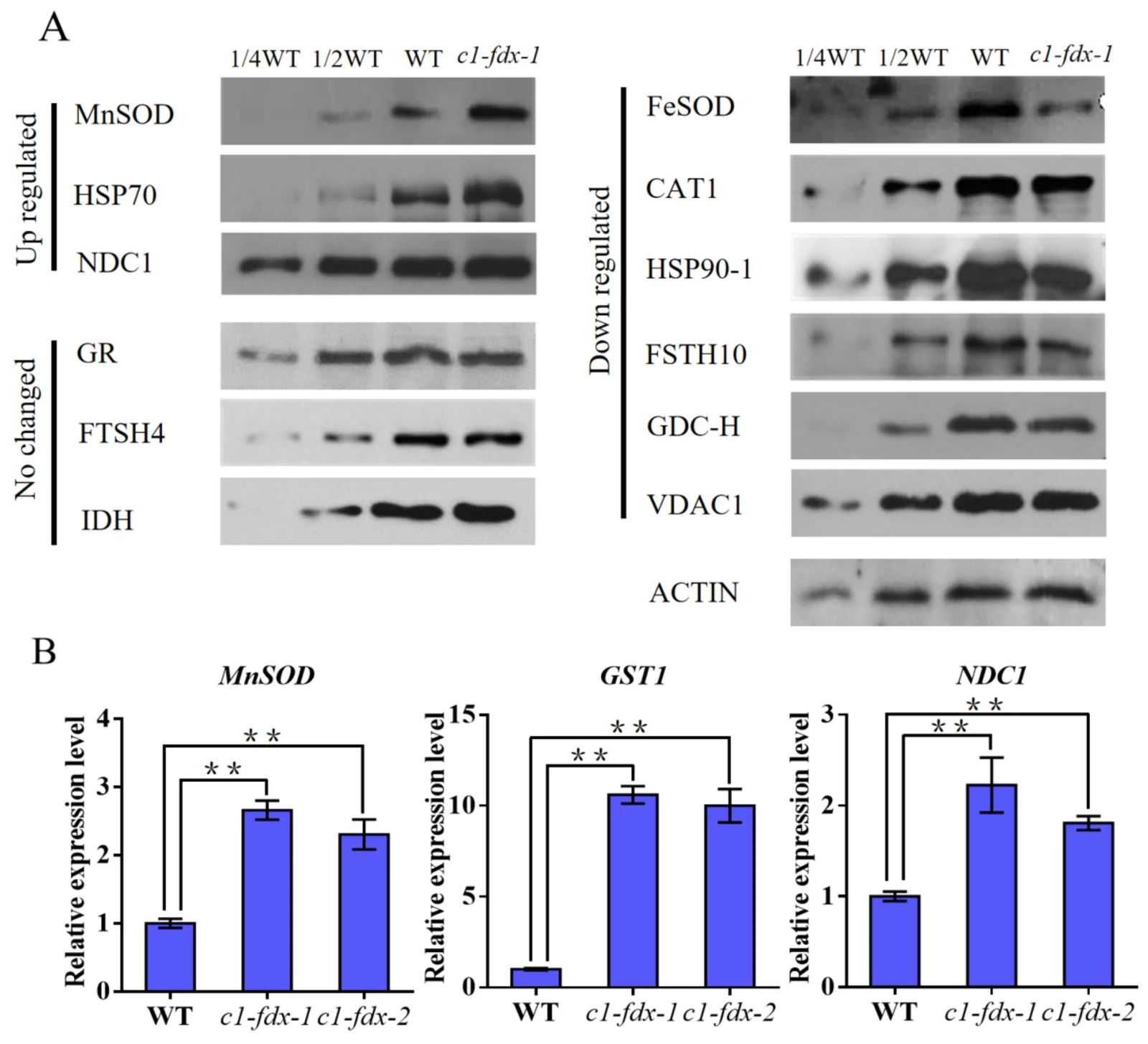
**Loss of Arabidopsis C1-FDX affects protein and gene levels.** (**A**) A total of 10 μg of mitochondrial membrane proteins from wild-type Arabidopsis and *c1-fdx* mutants was separated via SDS (sodium dodecyl sulfate) gel electrophoresis, blotted to the PVDF (Polyvinylidene Fluoride) membrane, and then probed with antibodies against the following proteins: MnSOD, manganese superoxide dismutase; HSP70, heat shock protein 70; NDC1, alternative NAD(P)H-ubiquinone oxidoreductase C1; GR, glutathione reductase; FTSH4, ATP-dependent zinc metalloprotease FtsH4; IDH, isocitrate dehydrogenase; FeSOD, Fe-dependent superoxide dismutase; CAT, catalase; HSP90-1, heat shock protein 90-1; FTSH10, ATP-dependent zinc metalloprotease; GDC-H, H protein of glycine decarboxylase complex; VDAC1, voltage-dependent anion-selective channel protein 1. ACTIN protein was used as the internal control. Here, 1/4 WT and 1/2 WT indicate protein loading amounts equivalent to one-quarter and one-half of the WT sample, respectively. (**B**) Quantitative real-time polymerase chain reaction (qRT-PCR) analysis of the gene expression of mitochondrial components in 3-day-old seedlings of the wild type and *c1-fdx* mutants. *Atubiquitin 10* was used as the internal control. Data are means ± SDs of three biological replicates. The wild type and each mutant were compared using one-way ANOVA followed by Dunnett’s test. Asterisks indicate significant differences relative to the WT plants. ** *p* < 0.01. *GST1*, *Glutathione S-transferase 1*.

To explore the mitochondrial antioxidant system’s response to ROS changes in *c1-fdx* mutants, we determined the protein abundance of a series of antioxidant enzymes, molecular chaperones, and proteases via Western blot and verified gene expression levels via qRT-PCR. For antioxidant proteins, the abundance of MnSOD was significantly upregulated in *c1-fdx* mutants at both the protein and transcriptional levels (Figure 4A,B). As the key enzyme specifically catalyzing O_2_^•−^ dismutation in the mitochondrial matrix, an increase in MnSOD activity will accelerate the conversion of excess O_2_^•−^ into H_2_O_2_ [11]. In contrast, the content of FeSOD (chloroplast-localized) and CAT1 had significantly decreased in mutants (Figure 4A). However, GR, an important component of the GSH redox cycle in terms of maintaining the overall mitochondrial redox environment, did not obviously change between the wild type and *c1-fdx-1* mutant (Figure 4A).

Regarding respiratory metabolism regulation, the protein and mRNA levels of alternative respiratory pathway component NDC1 were significantly higher in the *c1-fdx* mutants; this is a typical compensatory response of plants to the dysfunction of the main mitochondrial respiratory chain [30] (Figure 4A,B). Regarding mitochondrial-protein-homeostasis-related proteins, the molecular chaperone HSP70 accumulated in large quantities in mutants, a phenomenon that may be related to the blocked assembly of CI Fe-S subunits and the increased demand for de novo synthesis of CI components [31] (Figure 4A). However, the abundance of HSP90-1 and FTSH3/10 decreased markedly, while FTSH4 exhibited no significant changes (Figure 4A). Photorespiration-related proteins GDC-H exhibited only slight changes, indicating that C1-FDX deficiency mainly affects mitochondrial respiratory metabolism rather than photorespiration [32] (Figure 4A). The abovementioned results demonstrate that C1-FDX mutation comprehensively remodels the expression profiles of genes encoding mitochondrial functional proteins.

### 2.5. Antioxidant Enzymes Show Altered Activity in the c1-fdx Mutant

**Figure 5:**
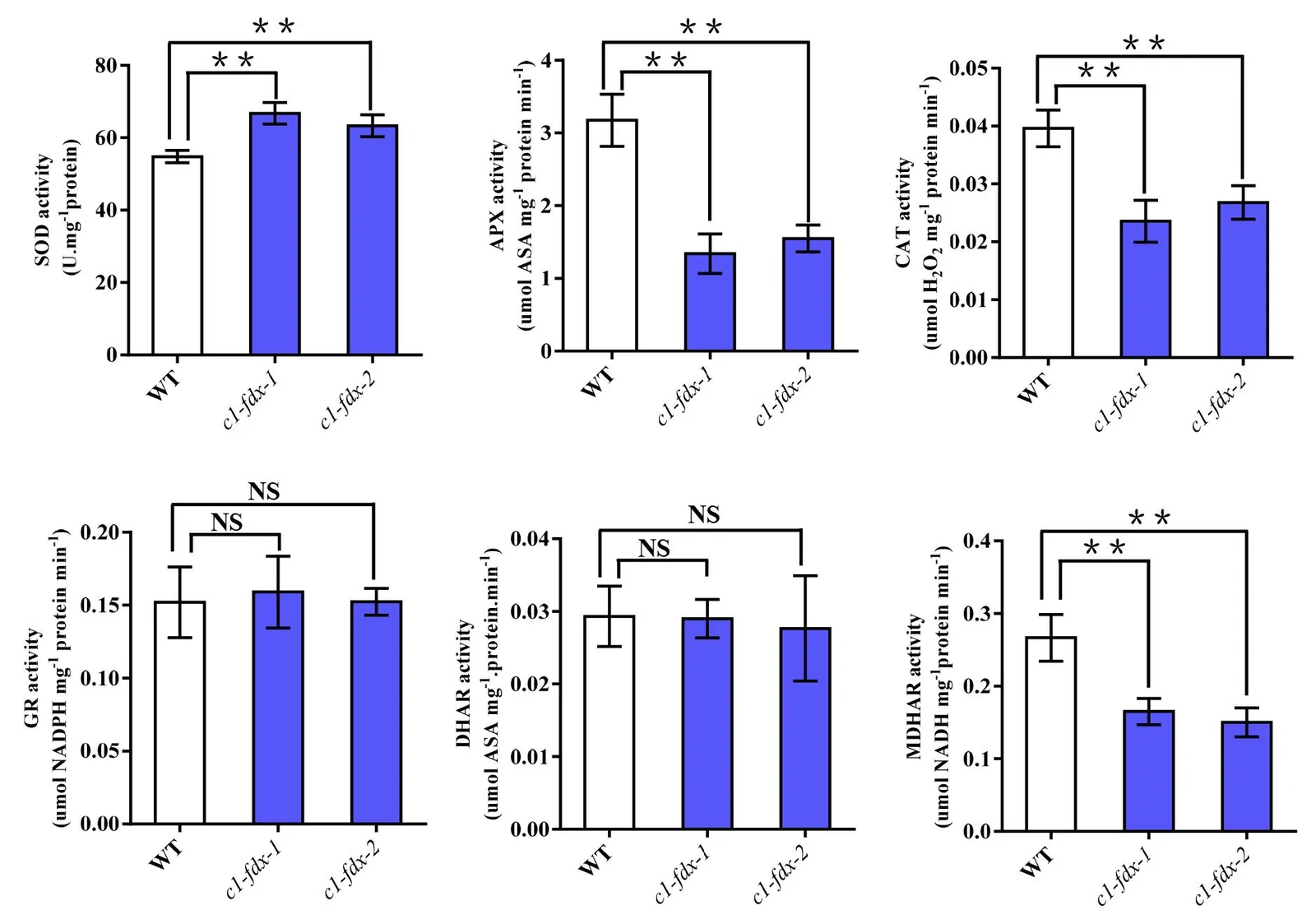
**Activity levels of antioxidant enzymes in wild-type Arabidopsis and the *c1-fdx* mutants in 3d old seedlings.** Superoxide dismutase (SOD); ascorbate peroxidase (APX); catalase (CAT); glutathione reductase (GR); dehydroascorbate reductase (DHAR); monodehydroascorbate reductase (MDHAR). Data are means ± SDs of three biological replicates. The wild type and each mutant were compared using one-way ANOVA followed by Dunnett’s test. Asterisks indicate significant differences relative to the WT plants. ** *p* < 0.01. NS, not significant.

To link protein abundance to catalytic function, we quantified the in vitro activity of core antioxidant enzymes in 3-day-old seedlings. Total SOD activity was significantly higher in *c1-fdx* mutants, possibly because of elevated MnSOD protein abundance (Figure 4A and Figure 5). CAT activity was markedly suppressed, matching the reduction in CAT1 protein abundance (Figure 4A and Figure 5). No significant differences in GR activity were detected between the WT and mutants, a finding consistent with unchanged GR protein levels (Figure 4A and Figure 5). The activity of APX, DHAR, and MDHAR displayed mild or non-significant alterations, indicating that the ascorbate–glutathione cycle was not the primary adaptive pathway activated in response to C1-FDX depletion (Figure 5).

## 3. Discussion

Mitochondrial CI is the largest and most complex multi-subunit complex in the plant respiratory chain. It undertakes the first step of electron transfer and is also the main source of intracellular ROS [1,5,13]. Numerous studies have shown that mutations in most structural subunits of CI will damage the integrity of the ETC, cause continuous electron leakage, and lead to the simultaneous accumulation of O_2_^•−^ and H_2_O_2_, triggering severe oxidative stress and multiple growth defects [12,33,34]. Our previous study showed that C1-FDX serves as a core structural component of complex I and is involved in the assembly of complexes I and V in Arabidopsis [8,9]. In this study, we found that the *c1-fdx* loss-of-function mutants presented a unique ROS phenotype: O_2_^•−^ massively accumulated, while H_2_O_2_ content reduced significantly (Figure 1). This distinct phenotype is completely different from the traditional CI subunit mutants, indicating that C1-FDX affects mitochondrial ROS homeostasis through a unique molecular mechanism that is unlike common CI structural subunits.

Our previous studies confirmed that the N-module of CI accumulates in large quantities in *c1-fdx* mutants, while the normal electron transfer from N-module to Q-module is interrupted [8]. The electrons trapped in the N-module cannot be transmitted downstream normally and continuously leak out, reacting with O_2_, which is the direct cause of O_2_^•−^ overproduction in mutants [14,15] (Figure 1). In addition, the blocked electron transport chain disrupts the cyclic oxidation and reduction of NADH and NAD^+^ (Figure 2). Our detection results confirm that the NADH content increased and NAD^+^ content decreased in *c1-fdx* mutants, leading to a sharply elevated NADH/NAD^+^ ratio (Figure 2). A high NADH/NAD^+^ ratio is a well-established positive regulator of O_2_^•−^ production at CI, further aggravating electron leakage and O_2_^•−^ generation [16]. Therefore, the combined effect of blocked CI assembly and an imbalanced NAD redox state leads to a significant accumulation of O_2_^•−^ in *c1-fdx* mutants.

In mitochondria, MnSOD is a matrix-specific antioxidant enzyme responsible for converting O_2_^•−^ into H_2_O_2_ [11,35]. Our Western blot and qRT-PCR results prove that the expression of MnSOD was significantly upregulated at both the protein and transcriptional levels in the *c1-fdx* mutants (Figure 4A,B). SOD activity also significantly increased in the *c1-fdx* mutants, likely because of the increase in MnSOD activity [35] (Figure 5). In plants, upregulation of MnSOD is a positive feedback response to excessive O_2_^•−^ accumulation (Figure S1). Enhanced MnSOD activity allows rapid scavenging of accumulated O_2_^•−^ and its conversion into H_2_O_2_ [11,18,35] (Figure S1). In theory, the large amount of H_2_O_2_ generated by O_2_^•−^ dismutation should lead to H_2_O_2_ accumulation in mutants, but our DAB staining results show the opposite trend. This contradiction might be attributable to iron metabolism and the Fenton reaction [2,20] (Figure S1).

As a conserved [2Fe-2S] ferredoxin, C1-FDX participates in the regulation of intracellular iron homeostasis, which is consistent with the function of mammalian mitochondrial FDX1/FDX2 [23]. After C1-FDX mutation, the Fe^3+^ content in roots increased (Figure 3). Fe^3+^ is an essential component of the Fenton reaction, and it can react with H_2_O_2_ to generate highly active hydroxyl radicals [18]. In *c1-fdx* mutants, the H_2_O_2_ newly generated by MnSOD might be continuously consumed by the Fenton reaction (Figure 2, Figure 4 and Figure S1). This cascade reaction consisting of ‘O_2_^•−^ → H_2_O_2_ → •OH’ eventually leads to a decrease in H_2_O_2_ content in mutant seedlings. This might be the core mechanism behind the opposite changes in O_2_^•−^ and H_2_O_2_ in *c1-fdx* mutants, and it also implies close crosstalk between ferredoxin, iron metabolism, and the Fenton reaction in plant mitochondria.

In terms of mitochondrial protein homeostasis and respiratory metabolism remodeling, we found that the alternative respiratory pathway protein NDC1 was upregulated in *c1-fdx* mutants (Figure 4). The main ETC function is impaired after a defect in CI assembly occurs, and plants activate the alternative respiratory pathway to bypass the blocked main chain, reduce electron leakage, and relieve the pressure of ROS burst, a classic adaptive compensatory mechanism in plants [6,7]. The molecular chaperone HSP70 accumulates in mutants, a phenomenon related to the increased synthesis of CI Fe-S subunits to compensate for defective CI assembly [31] (Figure 4). The downregulation of HSP90-1 and FTSH3/10 suggests that the protein degradation pathway in mitochondria is also affected after C1-FDX deficiency [36,37]. These results prove that C1-FDX has extensive regulatory effects on mitochondrial protein metabolism and the corresponding respiratory network, and these effects are not limited to ROS and iron metabolism.

Most studies have focused solely on ferredoxins’ functions as electron carriers or Fe-S cluster assembly factors in isolation [8,9,22,24,28]. This study focuses on C1-FDX and potentially links plant mitochondrial ferredoxin with the Fenton reaction and ROS dynamic balance, expanding the functional spectrum of plant mitochondrial ferredoxins and providing a new perspective for analyzing the complex regulatory network of mitochondrial CI. A limitation of this study is the lack of in vivo Fenton reaction activity measurement. In the future, researchers could test whether exogenous iron chelators rescue mutant ROS defects. C1-FDX may mediate stress tolerance via iron–ROS crosstalk in crops, a speculation requiring further investigation.

## 4. Materials and Methods

### 4.1. Plant Materials and Growth

The Arabidopsis *c1-fdx* mutants were generated using the CRISPR-Cas9 system described in our previous study [8]. *c1-fdx-1* and *c1-fdx-2* have a single base “T” or “G” insertion at +338 bp in the coding region, respectively, resulting in a frameshift and a translation stop codon next to the insertion [8]. Arabidopsis Columbia-0 (Col-0), *c1-fdx-1* and *c1-fdx-2* mutant seeds were surface-sterilized in 6.3% (*v*/*v*) NaClO containing 0.02% (*v*/*v*) Triton-X 100 for 15 min, washed 3 times in distilled water. For histochemical staining, total RNA extraction, and antioxidant enzyme isolation, surface-sterilized seeds were plated onto Murashige and Skoog (MS) medium containing 1% (*w*/*v*) sucrose and 0.8% (*w*/*v*) agar, and treated in the dark at 4 °C for 3 days. Then, the resulting seedlings were grown in growth chambers under a long-day photoperiod with 16 h of light (80 μmol quanta m^−2^ s^−1^, 22 °C) for 3 days. For mitochondrial isolation, 500 μg surface-sterilized seeds per sample were cultured in 150 mL liquid 1/2 MS medium containing 0.5% (*w*/*v*) sucrose. Seed stratification was performed on an orbital shaker at 45 rpm in darkness at 4 °C for 3 days [38]. The flasks were then transferred to 25 °C, and etiolated seedlings were cultured in the same liquid 1/2 MS medium in darkness for another 3 days.

### 4.2. In Situ Histochemical Staining of Ros and Iron Ions

We used 3,3-diaminobenzidine (DAB) staining (Beyotime, Shanghai, China) to detect the in situ accumulation of H_2_O_2_ and nitroblue tetrazolium (NBT) staining (Beyotime, Shanghai, China) to visualize the O_2_^•−^ distribution, referring to previously reported methods with minor modifications [39,40]. Three-day-old uniform seedlings were selected and immersed in freshly prepared staining solution. For DAB staining, seedlings were incubated in 2.5 nmol/L DAB solution (prepared with 50 mmol/L of phosphate buffer, pH 7.2) in the dark for 8 h at room temperature. For NBT staining, seedlings were incubated in 0.05% (*m*/*v*) NBT solution (50 mmol/L of phosphate buffer, pH 7.4) in the dark for 12 h. The staining intensity of DAB and NBT was quantitatively measured using Image-pro plus version 6.0 (Media Cybernetics, Rockville, MD, USA) software.

Prussian blue staining (Solarbio, Beijing, China) for total iron localization was performed to visualize the Fe^3+^ distribution in seedling roots. Three-day-old Arabidopsis seedlings were immersed in fresh staining solution containing 2% (*w*/*v*) potassium ferrocyanide and 2% (*v*/*v*) hydrochloric acid and incubated in the dark at room temperature for 6 h. After staining, the seedlings were rinsed 3 times with distilled water to remove residual staining reagent. Root iron deposition was directly photographed under a stereomicroscope. Meanwhile, absorbance was measured using leaching liquor, and photographs were taken. Three independent biological replicates were established for staining experiments.

### 4.3. Quantitative Real-Time Pcr (Qrt-Pcr)

Total RNA was extracted from three-day-old seedlings (Vazyme, Nanjing, China) and digested using DNase I (New England Biolabs, MA, USA) to remove residual DNA contamination. The RNA was quantified using an Agilent RNA 2100 bioanalyzer (Agilent, Santa Clara, CA, USA). Reverse transcription was performed to synthesize first-strand cDNA (TransGen Biotech, Beijing, China). qRT-PCR was carried out using SYBR Green qPCR mix (Vazyme, Nanjing, China) and specific primers (Table S1) via a real-time fluorescence quantitative PCR instrument. *Atubiquitin 10* was used as the internal reference gene. The relative expression levels of target genes (*MnSOD*, *GST1*, and *NDC1*) were calculated using the 2^−ΔΔCt^ method. Each sample was set to have three biological replicates and three technical replicates.

### 4.4. Antioxidant Enzyme Activity

All antioxidant enzymes were extracted from 3-day-old seedlings, with all extraction procedures performed at 4 °C. The antioxidant enzyme activity was determined spectrophotometrically at 25 °C in accordance with previously described methods with minor modifications [41]. Specifically, superoxide dismutase (EC 1.15.1.1, SOD) activity was assayed in a 1 mL reaction mixture containing 50 mM potassium phosphate buffer (pH 7.8), 0.1 mM EDTA, 75 mM NBT, 13 mM methionine, and 16.7 mM riboflavin by monitoring the inhibition of NBT photochemical reduction at 560 nm. Ascorbate peroxidase (EC 1.11.1.7, APX) activity was measured in a 1 mL reaction system consisting of 50 mM potassium phosphate buffer (pH 7.0), 1 mM ascorbic acid (AsA), and 0.25 mM H_2_O_2_ via the decrease in AsA absorbance at 290 nm with an extinction coefficient of 2.8 mM^−1^ cm^−1^. Catalase (EC 1.11.1.6, CAT) activity was determined in 50 mM potassium phosphate buffer (pH 7.5) containing 0.1 mM H_2_O_2_ by detecting the decomposition of H_2_O_2_ at 240 nm (extinction coefficient = 0.04 mM^−1^ cm^−1^). Glutathione reductase (EC 1.6.4.2, GR) activity was quantified in a 1 mL reaction solution supplemented with 50 mM potassium phosphate (pH 7.8), 0.5 mM oxidized glutathione (GSSG), 5 mM MgCl_2_, and 0.15 mM NADPH by recording the NADPH oxidation-induced absorbance decline at 340 nm (6.2 mM^−1^ cm^−1^). Dehydroascorbate reductase (EC 1.8.5.1, DHAR) activity was assayed in 50 mM potassium phosphate buffer (pH 6.3) with 1 mM dehydroascorbate (DHA) and 2 mM reduced glutathione (GSH) by measuring the increase in absorbance at 265 nm (14 mM^−1^ cm^−1^) resulting from DHA regeneration. Monodehydroascorbate reductase (EC 1.6.5.4, MDHAR) activity was determined in a reaction mixture containing 50 mM potassium phosphate (pH 7.0), 0.2 mM NADH, 2.5 mM AsA, and 0.2 U ascorbate oxidase based on the decreased absorbance at 340 nm (6.2 mM^−1^ cm^−1^) due to NADH oxidation.

### 4.5. Mitochondrial Isolation and Protein Complex Extraction

All extraction operations of mitochondrial isolation were carried out at 4 °C to prevent protein denaturation and mitochondrial activity loss. Crude mitochondria were isolated from the etiolated seedlings as described previously by Chen et al. [8]. Briefly, etiolated seedlings were gently ground on ice in an extraction buffer (0.3 M sucrose, 5 mM tetrasodium pyrophosphate, 10 mM KH_2_PO_4_, pH 7.5, 2 mM EDTA, 1% (*w*/*v*) polyvinylpyrrolidone 40, 1% (*w*/*v*) BSA, 5 mM cysteine, and 20 mM ascorbic acid). The homogenate was centrifuged at 3000× *g* for 5 min at 4 °C, and the supernatant was centrifuged once at 18,000× *g* for 30 min at 4 °C. The pellets were resuspended in wash buffer (0.3 M sucrose, 1 mM EGTA, 10 mM MOPS-KOH, pH 7.2). After isolation, the concentration of total mitochondrial protein was determined using the Bradford method [42]. Mitochondrial membrane protein complexes were solubilized with 2% dodecylmaltoside (SigmaSaint Louis, MS, USA) for subsequent Western blot analysis [25].

### 4.6. Western Blotting

Mitochondrial proteins were separated via 12.5% sodium dodecyl sulfate–polyacrylamide gel electrophoresis (SDS-PAGE). After electrophoresis, proteins were electrically transferred to 0.45 μm Polyvinylidene Fluoride (PVDF) membranes (Millipore, Burlington, MA, USA). The membranes were blocked with 5% skim milk powder solution at room temperature for 1 h and then incubated with primary antibodies overnight at 4 °C. Primary antibodies against MnSOD, IDH, GDC-H, FeSOD, GR, HSP70, and HSP90-1 were used as described in previous studies [43]. Other primary antibodies targeting Arabidopsis mitochondrial proteins were purchased from PhytoAB (USA) and Agrisera (Agrisera AB, Vännäs, Sweden). After incubation with horseradish peroxidase (HRP)-conjugated secondary antibodies, protein signals were detected using ECL chemiluminescence reagents (Thermo Fisher Scientific, Waltham, MA, USA).

### 4.7. Detection of the Mitochondrial NADH and NAD^+^ Content

Mitochondrial NADH and NAD^+^ content was determined using a WST-8 NAD^+^/NADH assay kit (Beyotime, Shanghai, China) in accordance with previously described methods, with minor modifications [44]. Briefly, 200 μL (containing 500 μg of protein) of 3-day-old seedling mitochondria was transferred into 600 μL of enhanced NAD^+^/NADH extraction buffer on ice and then centrifuged at 12,000× *g* for 10 min at 4 °C. For total NAD^+^/NADH measurement, half of the supernatants were mixed with a dehydrogenase working solution and incubated at 37 °C for 10 min. Then, a reaction buffer (20 mM Tris-HCl, 200 μM WST-8, and 8 μM PMS, pH 9.0) was added and reacted at 37 °C for 30 min. Absorbance was measured at 450 nm by a Varioskan LUX microplate reader (Thermo FisherWaltham, MA, USA). For NADH detection, the remaining half supernatants were incubated at 60 °C for 10 min, and the absorbance was measured after the reaction. The standard curve was generated and measured on the same plate as the samples. The NAD^+^/NADH ratio was calculated using the standard curves for NAD^+^ and NADH.

### 4.8. Statistical Analysis

Each experiment was repeated three times independently. Statistical analysis was performed using GraphPad Prism v6.01 software. Quantitative data are shown as means ± SDs. The two experimental groups were compared using one-way ANOVA followed by Dunnett’s test. Statistical significance was set at *p* < 0.05.

## 5. Conclusions

We hypothesized that mitochondrial C1-FDX maintains plant mitochondrial redox and iron homeostasis by coordinating electron transport, antioxidant machinery, and the Fenton reaction. Loss-of-function *c1-fdx* mutants displayed a unique opposing ROS pattern: over-accumulation of O_2_^•−^ and a depletion in H_2_O_2_, driven by an increase in the NADH/NAD^+^ ratio that triggered CI electron leakage and tissue Fe^3+^ overload. Upregulated MnSOD converts O_2_^•−^ into H_2_O_2_, while surplus Fe^3+^ may continuously consume H_2_O_2_ via the Fenton reaction to form hydroxyl radicals, potentially explaining this contradictory ROS shift. C1-FDX depletion also remodels mitochondrial chaperones, proteases, and alternative respiratory proteins as compensatory responses. This work links plant mitochondrial ferredoxin to Fenton-mediated ROS remodeling, expanding known ferredoxin functions beyond electron transport and Fe-S cluster assembly.

## Data Availability

The original contributions presented in this study are included in the article/Supplementary Materials. Further inquiries can be directed to the corresponding author.

## Supplementary Materials

The following supporting information can be downloaded at: https://www.mdpi.com/article/10.3390/ijms27209349/s1.
