# Supplementary material for: Mitochondrial C1-FDX Depletion Perturbs ROS Homeostasis and Iron Metabolism in Arabidopsis
Source: Int J Mol Sci. 2026 Oct 21;27(20):9349. doi: 10.3390/ijms27209349 (PMC13705397; doi:10.3390/ijms27209349)
Supplement: Supplementary file 1 [file ijms-27-09349-s001.zip › ijms-4592794-supplementary.pdf]

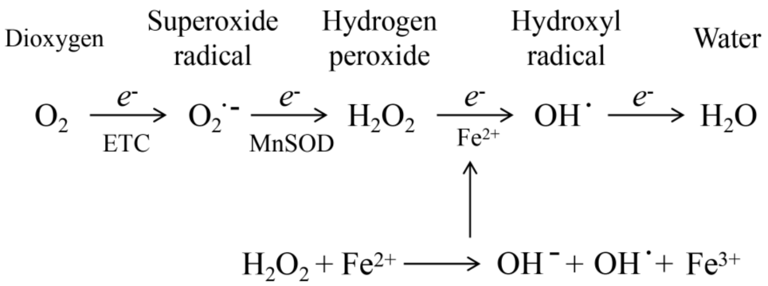

Figure S1. Schematic diagram of sequential ROS generation driven by mitochondrial electron transport chain and Fenton reaction.

Electron leakage from mitochondrial ETC reduces O_2 to produce superoxide anion ($O_2^{\bullet-}$). Matrix-localized MnSOD catalyzes $O_2^{\bullet-}$ dismutation to generate hydrogen peroxide (H_2O_2). The Fenton reaction utilizes H_2O_2 to generate the highly reactive hydroxyl radical (•OH). This •OH can be transformed into water by accepting an electron. The schematic representation is based on the works of Mittler [2] and Sweetlove and Foyer [20].

Table S1. Primer sequences used in this study.

Primer	Forward primer （5′-3′）	Reverse primer （5′-3′）	Purpose
MnSOD	TCCTGATCTTCCGTATGC	GCCGTTGAACTTGATGAG	RT-qPCR
GST1	CAGGCTTGGTGAGTCCAAGT	AGCAACCCAAGCACTCACAT	
NDCI	CAACAGTGGCACCACAGAGA	AGCCACCACCTAGAATGCAC	
Atubiqutin 10	GCCAAGATCCAGGACAAGG	CGCAGGACCAAGTGAAGAG	
